# Incidence and Bayesian Mapping of Myeloid Hematologic Malignancies in Sardinia, Italy

**DOI:** 10.1177/10732748231202906

**Published:** 2023-10-25

**Authors:** Giorgio Broccia, Jonathan Carter, Cansu Ozsin-Ozler, Sara De Matteis, Pierluigi Cocco

**Affiliations:** 1 Department of Haematology and Bone Marrow Transplants, Hospital A. Businco, Cagliari, Italy; 2 2706 University of Coventry, Coventry, UK; 3 Department of Paediatric Dentistry, Faculty of Dentistry, 37515 Hacettepe University, Ankara, Turkey; 4 Department of Medical Sciences and Public Health, 3111 University of Cagliari, Monserrato, Italy; 5 Division of Public Health, Health Services Research & Primary Care, Centre for Occupational and Environmental Health, 5292 University of Manchester, Manchester, UK

**Keywords:** myeloid malignancy, leukemia, myeloid, acute, myelodysplastic syndromes, incidence, Bayes theorem, socio-economic factors, livestock

## Abstract

**Background:**

The epidemiology of myeloid hematologic malignancies in Italy has been poorly investigated.

**Methods:**

We used a validated database of 1974-2003 incident cases of hematologic malignancies among the resident population (all ages) of Sardinia, Italy, to describe the incidence of myeloid malignancies overall (N = 4389 cases) and by subtype. We investigated the time trend of acute myeloid leukemia (N = 1227 cases), chronic myeloid leukemia (N = 613 cases), and myelodysplastic syndrome (N = 1296 cases), and used Bayesian methods to explore their geographic spread, and Poisson regression analysis to estimate their association with environmental and socio-economic factors.

**Results:**

The annual standardized (world population) incidence rate (IR) of myeloid malignancies over the study period was 6.5 per 100,000 (95% CI 6.2-6.7). Myelodysplastic syndromes were the most prevalent subgroup (IR = 1.7, 95% CI 1.5-1.8). Incidence of all myeloid malignancies combined increased sharply during the study period with an annual percent change (APC) of 10.06% (95% CI 9.51-10.61), 19.77% for myelodysplastic syndromes (95% CI 19.63-19.91), and 3.18% (95% CI 2.99-3.37) for acute myeloid leukemia. Chronic myeloid leukemia did not show an upward trend. Apart from sporadic excesses in small rural communities and the major urban area, there was no evidence of spatial clustering. The risk of myeloid malignancies increased with increasing prevalence of sheep breeding.

**Conclusions:**

Our results might prompt further research on the local genetic and environmental determinants of myeloid hematologic malignancies.

## Highlights

• Incidence of myeloid neoplasms, acute myeloid leukemia, and myelodysplastic syndrome, but not chronic myeloid leukemia, increased in 1974-2003 in the Italian region of Sardinia.
• Using Bayesian techniques, we identified 5 communes with an elevated posterior probability of AML and two of MDS. The risk of myeloid neoplasms was associated with urban residence and the size of sheep breeding farms.
• Our results highlight the need for extending cancer registration to the whole region and might help locating the hematology units to meet the patients’ needs more efficiently.

## Introduction

Time trends and geographic variation in the incidence of myeloid malignancies (MYEL) suggest a role of environmental factors. The descriptive epidemiology of MYEL in Europe showed age-adjusted incidence rates (IRs) 9.7 × 100,000/year, ranging from 1.1 for chronic myeloid leukemia (CML) to 3.6 for acute myeloid leukemia (AML) and a prevalence of men in both.^1^ Similar results were reported in the United Kingdom^2^ and in Canada, where the AML incidence increased sharply in 1992-2010, with the highest rates in 5 industrial cities in Ontario.^3^ The same upward trend in AML but not CML incidence was reported to have occurred in Denmark from 1943 to 2003^4^ and in Algeria from 2006 to 2010 where the central part of the country was particularly affected.^5^ In 1990-2019, CML incidence decreased at the global level, with an estimated annual percent change (APC) of -1.04, and a sharper decrease in mortality and disability-adjusted-life-years (DALY).^6^ Among other myeloid neoplasms, myelodysplastic syndromes (MDSs) were so defined for the first time by the French-American-British Working Group in 1976^7^ as a precursor stage for AML; only a few studies explored its epidemiological features.^1,8,9^ A large multicentre Italian study of hematological malignancies did not observe a geographic pattern in the AML incidence among the participating areas.^10^ However, no studies have evaluated the time trend of specific MYEL neoplasms in Italy thus far.

In this paper, we explored the incidence of MYEL and its subtypes over 3 decades using the 1974-2003 database of incident hematological cancer in the population of Sardinia, Italy. This Mediterranean island is known for its genetic peculiarities^11^ due to millennial geographic isolation and selective pressure from malaria. We also investigated the time trend, geographical distribution, and possible socio-economic and environmental determinants of MYEL, AML, CML, and MDS.

## Material and methods

The database of hematological cancer used for this analysis was previously described.^12^ Briefly, to overcome the difficulties created by the unavailability of a Cancer Registry, by conducting an active search with the collaboration of all the regional health authorities, the chief hematologist of the Cagliari Oncology Hospital created a database of all incident hematological cancers diagnosed in 1974-2003 among the Sardinian population of both sexes and any age.^12^ This database was subsequently validated by comparison with mortality and hospitalization data,^13^ and, limited to the last decade and the northern part of the region, with Cancer Registry records.^14^ The total entries were 14,744. We abstracted the data of 4389 MYEL cases, including 1711 women and 2678 men, grouped according to the Hematologic Malignancies Research Network classification,^15^ and comprising 1227 AML cases, 613 CML cases, 1296 MDS cases, 203 cases of idiopathic myelofibrosis, 240 cases of polycythemia vera, 677 cases of essential thrombocythemia, and 133 cases including 12 cases of systemic mastocytosis and 121 unspecified MYEL. This paper focuses on the analysis of time trends and geographic distribution of MYEL, AML, CML, and MDS. Consistent with the 2008 update of the WHO classification of neoplasms of the hematopoietic and lymphoid tissue,^16^ the AML cases incorporated all the International Classification of Diseases for Oncology-3 (ICDO-3)^17^ codes within this group: 9861/3 (AML NOS); 9865/3, 9866/3, 9869/3, 9871/3, 9877/3, 9878/3, 9879/3, 9896/3, 9897/3, 9920/3 (AML with recurrent genetic abnormalities); 9895/3 (AML with myelodysplasia related changes); 9920/3 (therapy-related AML); and 9866/3 (acute promyelocytic leukemia). CML cases included the ICDO-3 code 9863/3 and 9875/3, and MDS the codes 9980/3, 9982/3, 9983/3, 9984/3, 9985/3, 9986/3, 9987/3, 9989/3, 9991/3, 9992/3. However, the database did not contain enough details to number the cases by subtype within each group. Besides, to maintain diagnostic consistency over the years, we refrained from using the additional clinical and bio-molecular information that became available to distinguish more specific disease entities. Residence at the time of diagnosis was missing for 180 cases (4.1%); these contributed to the age- and gender-specific regional rates but not to the analysis of environmental risk factors.

To allow the international comparability of our data, we calculated the age- and gender-standardized incidence rates (IRs) in the Sardinian population over the 30-year period using both the World Health Organization (WHO) and the European standard population for each diagnostic group of interest. To calculate the standardized annual IR for each of the 356 autonomous Sardinian municipalities (communes) existing in 1974, we applied the 1974-2003 regional rates to the person-years in the corresponding age and gender strata of the local population. Those municipalities that acquired autonomy in the subsequent years were kept incorporated with that of origin to preserve across-time comparability between rates. We calculated the time trend of the regional annual IR in 1974-2003 with the best fitting regression equation, as determined by the *R*^2^ value, and the average APC and its 95% confidence interval according to Fay MP et al.^18^

The geographical distribution of the probability of incident cases of MYEL, AML, CML, and MDS was explored using a Bayesian approach and plotted on the map of their territorial borders. Such maps are made publicly available by the Italian Institute for Statistics (ISTAT) (https://www.istat.it/it/archivio/104317) under the Creative Commons BY 3.0 IT License. Appendix 1 describes in detail the Bayesian methodology we adopted. Briefly, we arbitrarily defined a critical threshold of .999 to identify the communes in which the posterior probability of the observed number of cases was on the extreme right of the curve of its distribution, that is, less likely explained by chance.

We calculated the probability of exceeding the critical IR of each diagnostic group using bespoke python code (https://bespokesynth.github.io/BespokeSynthDocs/python.html). The ratio between the probability of the alternative hypothesis *H*_1_ vs that of the null hypothesis *H*_0_, calculated from the same posterior probability curve, is the likelihood ratio, which follows its own probability distribution. For each MYEL subgroup, based on the quintiles of its distribution, we plotted such probability on the regional map using the following color scale for the area of each commune: white ≤.165, light gray .166-.335, medium-light gray .336-.50, medium-dark gray .501-.80, and dark gray .801-.95. The communes associated with a probability >95% had a black shade.

We used Poisson regression analysis to calculate the relative risk (RR) and its 95% confidence interval (95% CI) associated with possible determinants of MYEL, AML, CML, and MDS, with the lowest quantile or the unexposed category as the reference. We considered the following covariates: proportion of inhabitants aged ≥75 years, the ISTAT deprivation index (http://istat.it), the distance from the nearest hospital, the natural radiation background as quintiles of the probability of α-emission from radon daughters above the threshold of 300 Bq/m^3^ (≤5%, 6%-10%, 11%-20%, 21%-30%, and 31% or more),^19^ the geology of the local territory,^1,20^ and the urban/rural type of commune. The urban/rural covariate was defined for each commune based on 5 community services (administrative, educational, health, judicial, and religious) that would daily attract commuters from the surrounding area. The list of communes bordering large industrial or military areas or where cork harvesting or mining were prevalent industries was modified from Biggeri et al.^21^ We also used information on per capita livestock (cattle, sheep, and goat farms) per commune^22^ to explore the link with potential exposure to zoonotic agents.

In the univariate analysis, socio-economic deprivation and distance from the nearest hospital were unrelated to the incidence of MYEL or its specific subtypes. Therefore, we explored risk associated with environmental exposures, with reference to the lowest or the null category, or the quaternary marine deposits geology. The analysis was conducted using SPSS® version 20.0. The Ethics Committee of the University Hospital of Cagliari approved the use of the 1974-2003 database of incident hematological malignancies in Sardinia for scientific purposes, in agreement with the Code of Ethics of the World Medical Association (Declaration of Helsinki), on December 18, 2019 (protocol No. PG 2019/18070).

## Results

**Figure 1.**
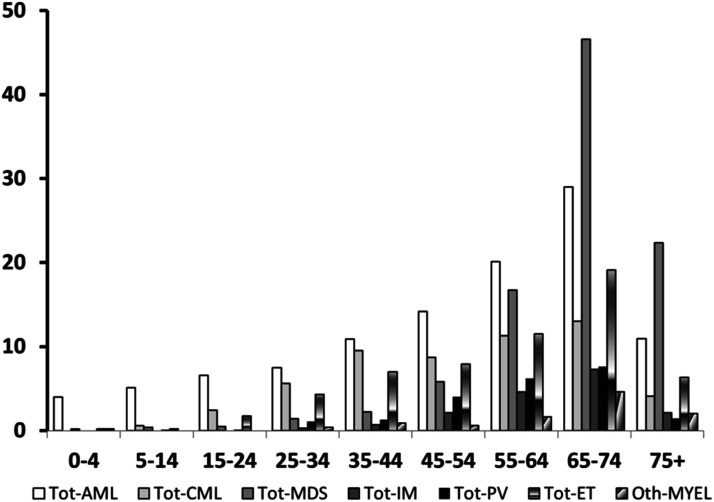
Age distribution of the prevalence of myeloid malignancies, acute and chronic myeloid leukemia, myelodisplastic syndromes, idiopathic myelofibrosis, polycythemia vera, essential thrombocythemia, and other myeloproliferative neoplasms. To preserve the comparability across age-groups of different size, the prevalence of each diagnostic group in each age class was divided by the corresponding number of years.

The average age at diagnosis of the 4389 MYEL cases was 62.6 years (SD 18.13), with male cases (No. = 2,678, mean 63.5, SD 17.64) slightly older than females (No. = 1,711, mean 61.3, SD 18.80, *P* < .0001). The mean age at diagnosis was similar for AML (mean = 56.6, SD 21.46) and CML (mean = 56.2, SD 18.23), and it was highest for MDS (mean = 71.3, SD 12.23) and idiopathic myelofibrosis cases (mean = 66.6, SD 11.84). Male cases prevailed in all subtypes but polycythemia vera (Table 1). As we had age classes of different size (5-year age class for 0-4 years, 10-year age class up to 74 years, and all ages above 75), to make their frequency distribution within the diagnostic groups comparable by type of myeloid malignancy we divided the number of cases in each age class by the number of years in it (Figure 1).

**Table 1.** Linear Regression Coefficients Describing the Time Trend of Incidence of Myeloid Malignancies by Age Group and Sex.

Diagnosis	No. cases	Age at diagnosis Mean (SD)	Incidence rate Sardinian population × 10^−5^ (95%CI)	Standardized incidence rate world population × 10^−5^ (95% CI)	Standardized incidence rate European population × 10^−5^ (95% CI)
Myeloid malignancies (all)					
Men	2678	63.5 (17.64)	11.2 (10.8-11.7)	8.1 (7.8-8.5)	17.0 (16.5-17.6)
Women	1711	61.3 (18.80)	7.0 (6.7-7.3)	4.8 (4.5-5.1)	8.8 (8.4-9.2)
Total	4389	62.6 (18.13)	9.1 (8.8-9.4)	6.5 (6.2-6.7)	12.9 (12.6-13.2)
Acute myeloid leukemia					
Men	726	57.6 (21.65)	3.0 (2.8-3.3)	2.4 (2.2-2.6)	4.3 (4.2-4.6)
Women	501	55.2 (21.15)	2.0 (1.9-2.2)	1.6 (1.4-1.7)	1.2 (1.1-1.4)
Total	1227	56.6 (21.46)	2.5 (2.4-2.7)	2.0 (1.8-2.1)	3.4 (3.2-3.5)
Chronic myeloid leukemia					
Men	379	57.0 (18.45)	1.6 (1.4-1.8)	1.2 (1.1-1.4)	2.2 (2.0-2.4)
Women	234	55.0 (17.84)	1.0 (.8-1.1)	.7 (.6-.8)	1.1 (1.0-1.3)
Total	613	56.2 (18.23)	1.3 (1.2-1.4)	1.0 (.9-1.1)	1.7 (1.5-1.8)
Myelodisplastic syndromes					
Men	856	71.7 (10.96)	3.6 (3.4-3.8)	2.3 (2.1-2.5)	6.1 (5.8-6.4)
Women	440	70.7 (14.37)	1.8 (1.6-2.0)	1.0 (.9-1.1)	4.0 (3.5-4.6)
Total	1296	71.3 (12.23)	2.7 (2.5-2.8)	1.7 (1.5-1.8)	4.3 (4.1-4.4)
Essential thrombocythemia					
Men	347	61.5 (17.05)	1.5 (1.3-1.6)	1.1 (.9-1.2)	2.1 (2.0-2.3)
Women	330	60.3 (17.66)	1.3 (1.2-1.5)	.9 (.8-1.0)	1.7 (1.5-1.8)
Total	677	60.9 (17.35)	1.4 (1.3 - 1.5)	1.0 (.9 - 1.1)	1.9 (1.8-2.0)
Idiopathic myelofibrosis					
Men	126	67.2 (10.54)	.5 (.4-.6)	.4 (.3-.5)	.8 (.7-.9)
Women	77	65.6 (13.73)	.3 (.2-.4)	.2 (.1-.3)	.4 (.3-.5)
Total	203	66.6 (11.84)	.4 (.4-.5)	.3 (.2-.3)	.6 (.5-.7)
Polycythemia vera					
Men	154	60.9 (13.43)	.6 (.5-.7)	.5 (.4-.6)	.9 (.8-1.0)
Women	86	63.0 (13.67)	.4 (.3-.4)	.2 (.2-.3)	.4 (.4-.5)
Total	240	61.7 (13.52)	.5 (.4-.6)	.4 (.3-.4)	.7 (.6-.8)

For each subtype, the cases tended to increase up to age 74 and declined afterward; AML was the only myeloid malignancy diagnosed before age 5.

### Time Trend

Table 1 shows the number of MYEL cases by gender and histology and the respective age-adjusted IRs in the total population and by gender, with the world population and the European population as the standard for wider comparability with international data.^2,23^ The world standardized IR of all MYEL combined was 6.5 per 100,000 (95% CI 6.2-6.7), varying from 2.0 (95% CI 1.8-2.1) for AML to .3 (95% CI .2-.3) for idiopathic myelofibrosis.

**Figure 2.**
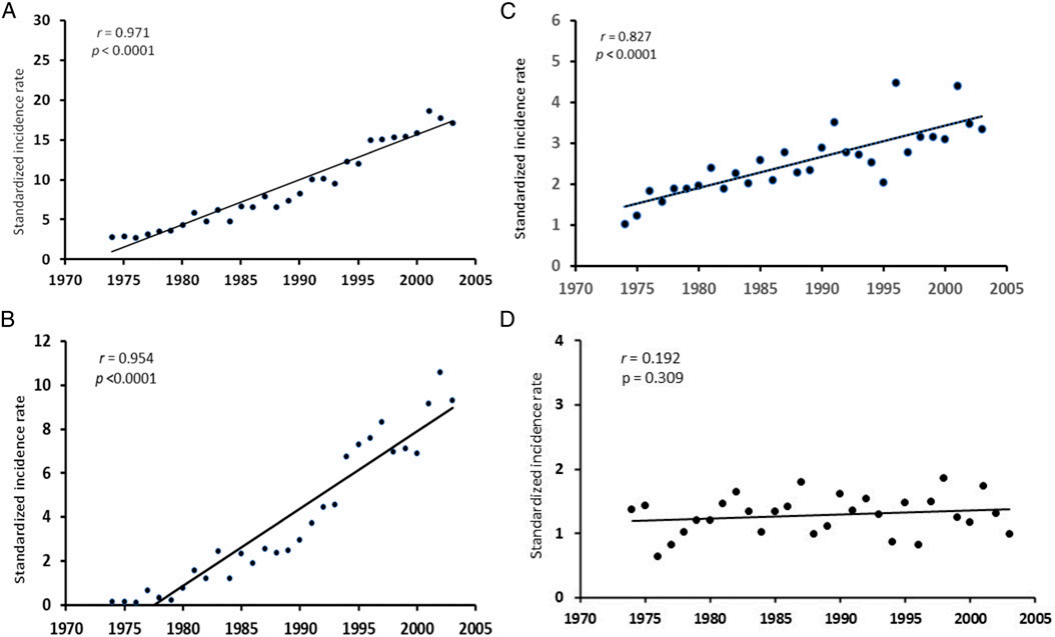
1974-2003 incidence rate of myeloid malignancies (A), myelodysplastic syndromes (B), acute myeloid leukemia (C), and chronic myeloid leukemia (D), (all ages, both sexes) in Sardinia, Italy.

Figure 2 shows the time trends of MYEL, AML, CML, and MDS in the total population. MYEL incidence increased linearly along the study period by .57 per 100,000 per year on average (APC: 10.06%, 95% CI 9.51-10.61) (Figure 2(A)), mainly due to a sharp upward trend in MDS incidence (APC: 19.77%, 95% CI 19.63-19.91) (Figure 2(B)). The APC was 3.18% for AML (95% CI 2.99-3.37) (Figure 2(C)), while CML did not vary along the study period (Figure 2(D)). Trends were consistent by sex, although more pronounced in men for AML and MDS (not shown in the tables). After plotting the residuals vs the predicted annual values,^24^ we did not detect any sudden change in the AML slope. Instead, the residual plot for MDS identified a join-point in 1990, indicating a sudden change in the frequency of diagnosis of the disease from that year onwards, most likely due to diagnostic advancements.

### Spatial Distribution of Myeloproliferative Neoplasms

**Figure 3.**
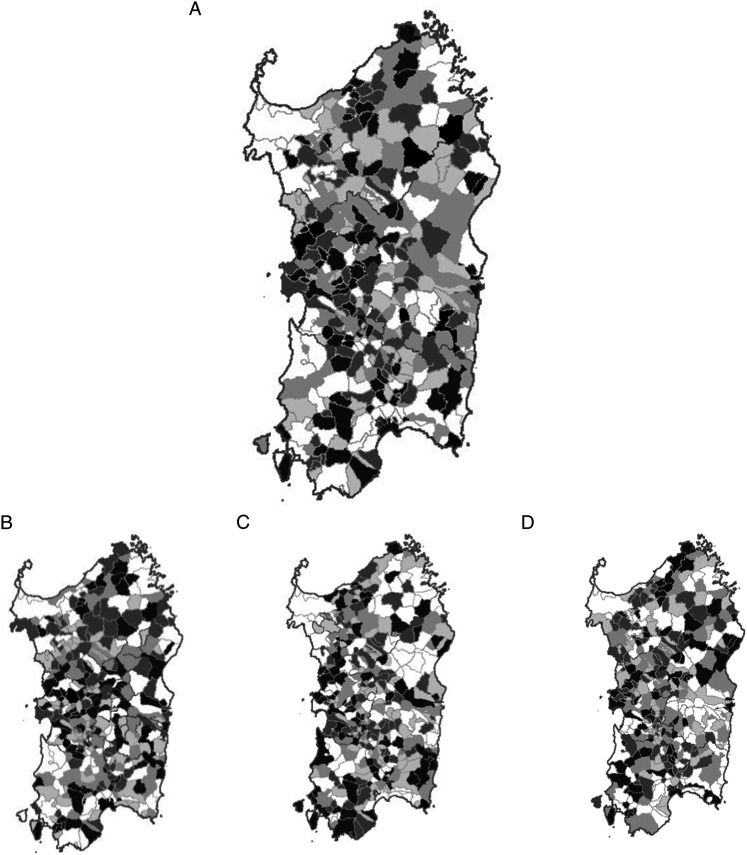
Map of 1974-2003 incidence of myeloid malignancies (A), acute myeloid leukemia (B), chronic myeloid leukemia (C), and myelodisplastic syndromes (D) in Sardinia, Italy. Color scale: white *P* ≤ .165, light gray *P* = .166-.335, medium-light gray *P* = .336-.50, medium-dark gray *P* = .501-.80, dark gray *P* = .801-.95, and black *P* > 95%. The maps of the territorial borders at the commune level are available online from the Italian Institute for Statistics (ISTAT) under the Creative Commons BY 3.0 IT license (https://www.istat.it/it/archivio/104317).

Figure 3 shows the map of the probability of MYEL, AML, CML, and MDS occurrence above the critical threshold. Contrary to what we previously reported for non-Hodgkin’s lymphoma (NHL) and multiple myeloma (MM),^14,25^ MYEL, AML, CML, and MDS did not cluster in specific areas of the regional territory. Also, the communes with a high probability of an excess incidence did not overlap with those with a high probability of NHL or MM.

Communes exceeding the 95% probability of a posterior IR of AML above the critical threshold of its prior distribution were 5, none for CML, and 2 for MDS. These communes appear randomly spread over the Sardinian territory, with no evidence of clustering in specific areas. Eight AML cases occurred among the male residents in a small commune of less than 1500 inhabitants in central-eastern Sardinia vs 1.05 expected. There were no female cases. All showed up in 1989-91 among subjects 50-72 years old. This commune is located in a mountainous area in the eastern part of the region; its economy is based on agriculture and small-size livestock farms, especially sheep and goats. We have no clues about what might have generated this excess. AML cases exceeded the expectation in another 3 rural communes in central-western Sardinia, with 8 observed vs 2.8 expected, 6 vs 1.4, and 8 vs 2.6, respectively. Their economy is mainly agricultural, with diverse types of crop and livestock farms, a few small food processing, textile and apparel manufacturing, and construction businesses. The fifth commune is the largest urban area and the region’s capital; it is a major seaport, and a site for military and industrial settlements. Vehicular traffic is heavier there than in the rest of the region.

### Environmental Exposures and Socio-Economic Factors

Urban residence was associated with a modest excess risk of MDS. The risk of MYEL, but not the specific subtypes we investigated, was elevated in areas whose geology mainly consisted of metamorphic rocks, which result from the transformation of igneous, sedimentary, or other rocks under extreme heat, pressure, or other natural conditions. The probability of indoor radon levels above 300 Bq/m^3^ did not affect risk. The risk of MYEL and CML but not AML and MDS increased with increasing sheep breeding size (Table 2).

**Table 2.** Risk of Myeloid Malignancies, Acute and Chronic Myeloid Leukemia, and Myelodisplastic Syndromes in Relation to Increasing Quartile of Background Radiation, Geological Characteristics, Livestock, and Major Economic Activities in the Surroundings.

Environmental factors	Person-years	Myeloid malignancies			Acute myeloid leukemia			Chronic myeloid leukemia			Myelodysplastic Syndromes		
		Cases	RR	95% CI	Cases	RR	95% CI	Cases	RR	95% CI	Cases	RR	95% CI
Type of residence													
Rural	21 012 674	1983	1.00	Reference	548	1.00	Reference	319	1.00	Reference	565	1.00	Reference
Small towns	8 705 250	671	.92	.84-1.01	180	.86	.73-1.02	94	.77	.61-.97	198	1.02	.86-1.19
Urban	18 708 946	1555	1.02	.98-.05	468	1.04	.97-1.10	196	.88	.80-.96	443	1.07	1.00-1.14
Geological features													
Quaternary or subsequent marine deposits	32 452 466	2750	1.00	Reference	768	1.00	Reference	412	1.00	Reference	785	1.00	Reference
Basalt and other effusive volcanic rocks	4 663 262	469	.78	.60-1.02	151	.78	.48-1.26	65	.55	.26-1.16	131	.97	.60-1.57
Granite and intrusive volcanic rocks	4 840 702	429	.95	.89-1.01	119	.95	.84-1.08	55	.85	.71-1.01	129	1.00	.89-1.13
Metamorphic rocks	6 470 440	561	1.17	1.08-1.26	158	.96	.86-1.07	77	.98	.84-1.15	161	1.00	.90-1.12
Probability of indoor radon >300 Bq/m^3^													
<5%	9 726 062	917	1.00	Reference	304	1.00	Reference	113	1.00	Reference	263	1.00	Reference
5-9.9%	13,308 388	1183	.84	.68-1.04	321	.77	.53-1.14	183	1.26	.71-2.25	351	.77	.51-1.15
10%-19.9%	14 889 308	1262	.89	.82-.96	316	.74	.63-.85	194	.99	.80-1.23	362	.92	.80-1.06
20%-29.9%	6 588 218	475	.92	.87-.97	152	.86	.79-.95	63	.86	.74-.99	125	.92	.83-1.02
Cattle per resident													
None	32 666 894	2847	1.00	Reference	843	1.00	Reference	412	1.00	Reference	802	1.00	Reference
.03-.10	9 278 600	678	1.02	.93-1.13	194	.85	.70-1.03	106	1.02	.79-1.33	234	1.03	.85-1.24
≥.11	6 481 376	584	1.00	.95-1.05	159	1.00	.91-1.10	91	1.01	.89-1.15	170	.99	.90-1.08
≥30%	3 914 894	372	.98	.95-1.02	103	.93	.86-.99	56	.98	.88-1.08	105	.99	.92-1.07
Sheep per resident*													
< 1.43	33 210 636	2446	1.00	Reference	703	1.00	Reference	341	1.00	Reference	706	1.00	Reference
1.43-3.349	9 217 810	796	.77	.53-1.12	231	.57	.28-1.16	109	.49	.16-1.44	232	1.04	.52-2.06
3.35-7.60	5 826 018	604	1.45	.98-2.16	149	1.14	.54-2.43	105	1.82	.66-5.01	160	1.22	.57-2.61
≥ 7.61	3 569 754	363	1.29	.83-2.01	113	1.58	.70-3.57	54	1.24	.37-4.07	108	1.53	.64-3.63
Goats per resident													
< 1/100 residents	12 829 196	1148	1.00	Reference	356	1.00	Reference	144	1.00	Reference	328	1.00	Reference
1/100-1/10 residents	18 183 036	1470	.81	.74-.89	387	.71	.60-.83	216	.92	.71-1.17	432	.81	.69-.96
≥ 1/9 residents	17 414 638	1591	.94	.89-.99	453	.90	.82-1.00	249	.95	.82-1.10	446	.92	.83-1.10
Industries/mines													
No	28 941 840	2568	1.00	Reference	722	1.00	Reference	398	1.00	Reference	715	1.00	Reference
Yes	19 485 030	1641	1.04	.96-1.12	474	1.00	.86-1.15	211	.96	.77-1.18	491	1.15	1.00-1.34
Cork harvesting													
No	47 692 060	4140	1.00	Reference	1173	1.00	Reference	602	1.00	Reference	1191	1.00	Reference
Yes	734 810	69	1.15	.88-1.50	23	1.53	.98-2.40	7	.84	.38-1.87	15	.65	.34-1.22
Military settlements and shooting ranges													
No	40 381 272	3460	1.00	Reference	954	1.00	Reference	529	1.00	Reference	992	1.00	Reference
Yes	8 045 598	749	1.05	.95-1.16	242	1.22	1.02-1.47	80	.85	.65-1.12	214	.98	.80-1.19

*Note*. * test for trend (*P*-value): myeloid malignancies: *P* < .0001; acute myeloid leukemia: *P* = .068; chronic myeloid leukemia: *P* = .005; myelodisplastic syndromes: *P* = .063.

MDS was associated with proximity to industries and AML risk was elevated in the surroundings of military settlements. The concentration of cases among residents in the largest urban area of the region, which is also the site of important military settlements, spuriously generated this last finding: indeed, in rural communes, the RR associated with proximity to military settlements was 1.00 (95% CI .67-1.49), and it was .68 (95% CI .36-1.31) in small towns.

## Discussion

### Time Trend

Our results show that, in the region of Sardinia, the incidence of MYEL sharply increased annually by 10.06% over the 3 decades covered by our database. The increasing diagnoses of MDS from the late 1970s and the moderate upward trend in AML but not CML incidence were main contributors. The 2000-2002 annual standardized MYEL incidence in the European population was 9.73 per 100,000,^1^ and 15.1 in the United Kingdom in 2004-2009;^2^ it was 12.9 in our study. AML incidence in Sardinia was close to the European rate (3.4 vs 3.6 × 100,000), but CML and MDS were 55% and 2.4-fold higher, respectively.

We observed time trends consistent with those reported in Denmark for the years 1943-2003.^4^ Also, the 1975-2011 United States Surveillance, Epidemiology, and End Results (SEER) data^26^ and the 1999-2018 Republic of Korea Statistical Information Service (KOSIS) data^27^ reported a linear increase in the MYEL incidence, with a tendency to decline in recent years. MYEL and, specifically, AML also showed increasing trends in Canada^3^ but not Spain.^9^ Consistent with our observation, the Spanish report and a Finnish study^10^ described an increasing trend in MDS incidence with a tendency to decline in recent years. While our observation, extending from before to more than 2 decades after the publication of the French-American-British Working Group diagnostic criteria, might be due to the increasing number of diagnoses of a disease previously unrecognized as a malignancy, the report of continuing upward trends in the subsequent years suggests a real increase in incidence. On the other hand, as previously observed,^12^ the stable CML incidence might be explained by the appearance of symptoms requiring medical intervention, well-defined clinical and laboratory features, and constant adequate diagnostic capability over the study period.

### Spatial Distribution

We did not find clear evidence of MYEL clustering in specific areas within the regional territory. The few communes with a high probability of AML and MDS occurrence were scattered over the region and unrelated. Spatial clustering was reported in an area of a Canadian city polluted by the emissions from local oil refineries and chemical plants, where IRs of AML were more than 3-fold above the national average in 1992-2010. Consistent with our findings, 5 industrial cities in Ontario showed IRs higher than the national average.^3^ Also, the incidence of myeloid neoplasms in Spain showed striking differences between the contributing Cancer Registries.^9^

### Environmental Exposures and Socio-Economic Factors

Environmental exposures were explored as possible determinants of the geographic variation of heterogeneous combinations of hematological neoplasms, such as the use of chemical fertilizers and acute leukemia in Dagestan, Russia,^28^ pesticide use and hemolymphatic cancer in Quebec, Canada,^29^ arsenic contamination of groundwater and leukemia in the Ganges plain, India,^30^ and volatile organic compounds (VOC) contamination in the drinking water and leukemia in New Jersey, USA.^31^ Also, following an oil spillage in 2007, leukemia incidence increased annually by 9.5% among women in Korean county vs .6% in the whole country.^32^ Incidence of myeloid leukemia showed a weak correlation with gasoline consumption per km^2^ in Europe,^33^ and MDS incidence with airborne benzene level in a US cohort.^34^ Geological features but not radon levels and gamma radiation were associated with myeloid leukemia in an Italian study.^35^ On the other hand, ^222^Rn activity above 90 Bq/m^3^ was well correlated with an increase in leukemia incidence in an international study.^36^

Previous reports have raised concern about an elevated incidence of and mortality from myeloid leukemia among residents’ industrial areas in Italy and Canada, particularly children and young adults.^3,37^ Besides, following vast concern in the aftermath of the Balkan war and the use of depleted uranium (DU) weapons there, the Italian media raised claims of an excess incidence of hematologic malignancies around the military shooting ranges in Sardinia, assuming DU weaponry use for training purposes. A previous investigation of cancer hospitalization and mortality in areas bordering military settlements did not find an excess of all leukemias combined. However, the authors could not discriminate between the various forms of leukemia.^21^ In our study, the AML IR exceeded the critical threshold in the largest urban area and the capital of Sardinia. Proximity to military settlements was also associated with an elevated AML risk, but the excess vanished after excluding the largest urban area. As the same excess was not replicated in other urban centers, the AML risk associated with urban residence was only marginally increased. This analysis would suggest that specific conditions in the region’s capital and not the presence of military installations or shooting ranges in the territory was the risk factor. Residence in the proximity of industrial settlements was associated with the risk of MDS; airborne benzene levels were reportedly elevated in one of these areas and even more in the region capital due to heavy vehicular traffic^38^ which would be consistent with the previously mentioned US report.^34^ Contact with livestock might imply human transmission of biological agents potentially relevant in leukemogenesis, such as the bovine leukemia virus, the avian flu virus, and other implicated in several sheep diseases, such as *Chlamydia psyttaci*, the blue tongue, and the foot-and-mouth disease viruses.^39,40^ The results of a multicentre European case-control study observed a protective effect against diffuse large B-cell lymphoma among those who started occupational contact with livestock before age 12.^41^ An ecologic study reported an association between the incidence of acute lymphocytic leukemia and cattle density in Iowa counties plagued with bovine leukemia outbreaks.^42^ To the best of our knowledge, no studies addressed specifically the hypothesis of a link with myeloid neoplasms. We also observed an upward trend in risk of MYEL, and CML with increasing sheep but not goat or cattle breeding size. The occurrence of an epidemic of blue tongue decimated the flocks in Sardinia from the late 1990s onwards;^43^ further analytical studies should explore whether any link exists with MYEL risk in humans.

### Limitations

The senior hematologist who initiated the database and reviewed all the diagnoses did preserve the comparability of data collected over 3 decades. This is an advantage of our study, as the error would have been uniformly spread over time and geographically, thus preventing diagnostic bias. Besides, we only had partial access to the original slides, and the database did not include information on stage. Therefore, to preserve the comparability over the years, we could not use the limited, additional clinical and bio-molecular information that became available to explore specific disease entities matching the classification updates.

We used a database dating decades back; the trends and geographical spread of myeloid neoplasms might have changed since then. Still, it is a unique resource covering incidence of specific groups of myeloid neoplasms over 3 decades in a whole geographic area, isolated for millennia, with peculiar genetic features, allowing comparisons with other European and extra-European countries during approximately the same time frame. Besides, this database allowed us to explore the time trend and geographic distribution of specific myeloid neoplasm for the first time in Italy.

Especially in the first years of creating the database, the active search of incident cases might have increased the awareness of the local physicians and therefore the diagnoses of hematological malignancies. The concurrent upgrade of the diagnostic equipment, including the widespread availability of automatic cell counters and the equipment for the electrophoresis of serum proteins, in those years and the easier access to hospitals and specialist care from the late seventies might have also contributed. This might have varied geographically and by specific disease entity, thus contributing to creating the false impression of an upward trend and spatial inequality in the spread of specific hematological malignancies.^12^ Such reporting bias might have affected our findings on AML and MDS. On the other hand, the continuing upward trends in the following decades, the stability of CML incidence, and the consistency of our findings with similar reports from Spain and Finland^9,10^ would support a real increasing incidence of AML and MDS. Therefore, our results might have resulted from the combining effect of a true increasing incidence and the increased diagnostic capability over the years. Post-diagnosis relocation of the families seems unlikely, as excess cases also showed up in small towns and villages, independent on the distance from the hospitals with specialist onco-hematology units. The information on the commune of residence at diagnosis was missing for 4.1% (180/4389) MYEL cases, a proportion small enough to ensure reliable results.

We conducted an ecological study with the population of each commune and not the individual as the unit. Although the ecological fallacy might have masked possible associations or generated false ones,^44^ the geographic distribution and time trends in the incidence of neoplastic diseases can provide clues about the role of increasing or decreasing exposure to widespread risk factors, can reflect underlying changes in the diagnostic classification, and can measure the effectiveness of therapeutic advances.^45^ Therefore, while stressing the public health relevance of our findings, we recommend caution in interpreting the associations or lack of association with the environmental factors we were able to investigate.

### Conclusion

In conclusion, our results might prompt further research on the local genetic and environmental determinants of myeloid hematologic malignancies and make the case for extending the cancer registration to the whole region. At the same time, they might serve as the basis for planning a location of the Hematology units so to more efficiently meet the patients’ needs.

## Appendix

AML: Acute myeloid leukemia
APC: Annual percent change
CI: Confidence interval
CML: Chronic myeloid leukemia
DALY: Disability-adjusted-life-years
ET: Essential thrombocythemia
IM: Idiopathic myelofibrosis
IR: Incidence rate
ISTAT: Italian Institute for Statistics
MDS: Myelodysplastic syndrome
MM: Multiple myeloma
MYEL: Myeloid malignancies
NHL: Non-Hodgkin’s lymphoma
PV: Polycythemia vera
RR: Relative risk
U.S.A.: United States of America
VOC: Volatile organic compound
WHO: World Health Organization
